# Multimodality Molecular Imaging to Monitor Transplanted Stem Cells for the Treatment of Ischemic Heart Disease

**DOI:** 10.1371/journal.pone.0090543

**Published:** 2014-03-07

**Authors:** Zhijun Pei, Xiaoli Lan, Zhen Cheng, Chunxia Qin, Xiaotian Xia, Hui Yuan, Zhiling Ding, Yongxue Zhang

**Affiliations:** 1 Department of Nuclear Medicine, Union Hospital, Tongji Medical College of Huazhong University of Science and Technology, Hubei Province Key Laboratory of Molecular Imaging, Wuhan, China; 2 Department of PET Center, Taihe Hospital, Hubei University of Medicine, Shiyan City, Hubei Province, China; 3 Molecular Imaging Program at Stanford and Bio-X Program, Stanford University, Stanford, California, United States of America; Baker IDI Heart and Diabetes Institute, Australia

## Abstract

**Purpose:**

Non-invasive techniques to monitor the survival and migration of transplanted stem cells in real-time is crucial for the success of stem cell therapy. The aim of this study was to explore multimodality molecular imaging to monitor transplanted stem cells with a triple-fused reporter gene [TGF; herpes simplex virus type 1 thymidine kinase (HSV1-tk), enhanced green fluorescence protein (eGFP), and firefly luciferase (FLuc)] in acute myocardial infarction rat models.

**Methods:**

Rat myocardial infarction was established by ligating the left anterior descending coronary artery. A recombinant adenovirus carrying TGF (Ad5-TGF) was constructed. After transfection with Ad5-TGF, 5×10^6^ bone marrow mesenchymal stem cells (BMSCs) were transplanted into the anterior wall of the left ventricle (n = 14). Untransfected BMSCs were as controls (n = 8). MicroPET/CT, fluorescence and bioluminescence imaging were performed. Continuous images were obtained at day 2, 3 and 7 after transplantation with all three imaging modalities and additional images were performed with bioluminescence imaging until day 15 after transplantation.

**Results:**

High signals in the heart area were observed using microPET/CT, fluorescence and bioluminescence imaging of infarcted rats injected with Ad5-TGF-transfected BMSCs, whereas no signals were observed in controls. Semi-quantitative analysis showed the gradual decrease of signals in all three imaging modalities with time. Immunohistochemistry assays confirmed the location of the TGF protein expression was the same as the site of stem cell-specific marker expression, suggesting that TGF tracked the stem cells in situ.

**Conclusions:**

We demonstrated that TGF could be used as a reporter gene to monitor stem cells in a myocardial infarction model by multimodality molecular imaging.

## Introduction

Although significant progress has been made in coronary revascularization and atherosclerosis prevention, cardiovascular diseases are still a major cause of death. Many animal and clinical experiments have demonstrated that treating ischemic heart disease with transplanted bone marrow mesenchymal stem cells (BMSCs) is feasible and promising [1]–[6]. Although traditional techniques such as in situ hybridization, PCR and immunohistochemistry are widely used to analyze the distribution and migration of transplanted stem cells, they are in vitro or post mortem and obviously not applicable for in vivo studies. Therefore, using non-invasive techniques to monitor the survival and migration of transplanted stem cells in real-time is crucial for the success of therapy.

In the past decade, techniques to monitor transplanted stem cells have reached a new stage in which the biological progress of transplanted tissues and cells can be monitored in vivo at the molecular level. A variety of cutting edge molecular imaging techniques have been developed [7], [8]. Different imaging methods have their advantages and disadvantages. Radionuclide imaging is highly sensitive but suffers low spatial resolution. Magnetic resonance imaging (MRI) shows the highest soft tissue contrast, but has low sensitivity. Bioluminescence and fluorescence imaging have relatively high sensitivity and spatial resolution, but cannot image deep tissues. To address these issues, multimodality molecular imaging has been actively developed in recent years. Many of these multimodality imaging techniques, such as radionuclide/MRI and optical imaging, are further used for cell trafficking such as stem cell monitoring [9]–[11]. These studies indicate that multimodality imaging has advantages over single-modality imaging. Furthermore, considering multimodality imaging probes and image fusion techniques have been developed rapidly, multimodality imaging may find many important applications in clinical practice.

The triple fusion gene TGF [herpes simplex virus type 1 thymidine kinase (HSV1-tk), enhanced green fluorescence protein (eGFP) and firefly luciferase (Fluc)] was recently developed and applied to stem cell monitoring [12], [13]. We have shown similar results in a previous study [14]. However, TGF has not been used to monitor transplanted stem cells in a myocardial infarction model. Therefore, in our current study, we aimed to determine whether TGF can be expressed in a myocardial infarction model using three imaging methods, and the duration of possible TGF expression. We explored the feasibility of multimodality imaging to monitor the transplanted stem cells in rat models with ischemic heart disease.

## Materials and Methods

### Ad5-TGF plasmid construction and recombinant adenovirus packaging

The pCDNA3.1 plasmid carrying the TGF fusion gene under the control of the CMV promoter was kindly provided by Dr. Sanjiv Sam Gambhir, Stanford University. The plasmid was repackaged into a recombinant adenovirus (Ad5-TGF) that was amplified and purified. The virus titer of 1.26×10^10 ^TU/mL in 7 mL was determined by Vector Gene Technology Co., Ltd (Beijing, China).

### Isolation and culture of BMSCs, and Ad5-TGF infection of BMSCs

All studies were approved by the Institutional Animal Care and Use Committee (IACUC) at Tongji Medical College, Huazhong University of Science and Technology (HUST). All the specific animals were maintained in the barrier system room at 22±2°C with an alternating 12 h light/dark cycle, and were given food and water ad libitum throughout the study period. All procedures in the animal experiment were carried out in strict compliance with the Guideline of Laboratory Animals Ethics Committee of Tongji Medical College, HUST. MSCs were isolated from the femur and tibia of a 4-week-old healthy Sprague-Dawley (SD) rat supplied by the Experimental Animal Center of Tongji Medical College, HUST (Wuhan, China). The bone marrow was flushed out with DMEM-F12 (Gibco, Langley, VA) containing 15% fetal bovine serum (FBS) (Gibco, Langley, VA), and then the cells were seeded in six-well plates. Passage 3–5 BMSCs cultured in OPTI-MEM serum-free medium (Gibco, Langley, VA) were infected with Ad5-TGF. The required volume of Ad5-TGF was calculated by the specific multiplicity of infection (MOI) of the BMSC count. Cells were slowly shaken inside a 37°C incubator for 2 h, and they were then transferred into a 37°C/5% CO_2_ incubator for 48 h.

### Animal model

Thirty specific pathogen-free SD rats (200±25 g) were used to establish the animal model of acute myocardial infarction using previously published methods [15]–[18]. Rats were anesthetized with Isoflurane (Shanghai Abbott Laboratories, China) and fixed supinely on the operating table. The chest hair was shaved, and the skin was sterilized. A 2 cm incision was made at the left margin of the sternum in the third to fourth intercostal along the direction of the intercostal space. Deep and superficial fasciae were cut. The pectoralis major and serratus anterior muscles were separated to expose the ribs. After the bleeding stopped, the fourth intercostal muscle was separated, and the heart was quickly returned and the left anterior descending artery was ligated with a non-invasive suture. Each layer of the chest wall was sutured after the rat was able to breathe spontaneously.

BMSCs were infected with Ad5-TGF (MOI = 100) for 48 h. Rats (n = 22) that survived for 1 week after surgery were used for experiments. The method described above was used to extrude the heart. Ad5-TGF-infected BMSCs (5×10^6^ cells in 100 µl) were slowly injected into the myocardium of the left ventricular anterior wall at the far end of the coronary artery ligation. Rats were transplanted with uninfected BMSCs for the negative control group (n = 8). In the experimental group (n = 14), five rats were performed microPET/CT and bioluminescence imaging continuously, and the other nine rats were used to obtain fluorescence imaging.

All rats for the model preparing were put on heat preservation pads during the surgery and till to fully awake. Surgical wound was prevented to expand during the surgery, which in turn minimized post-operative pain and distress. Penicillin (10^5 ^U/kg, every 24 hours) was intramuscularly injected for 4–5 days after the surgery in order to prevent infection. All rats were allocated to individual cage post-operation, and bred with nutritious pellets. After surgery, body weight, heart rate, respiration and specific behaviors of rats were recorded every 24 hours for at least one week. Rats were sacrificed after multimodality imaging with overdose anesthetic drug (pentobarbital, 150 mg/kg). All the animal experimental procedures were carried out under the guidance of the veterinaries in Department of Experimental Animals, Tongji Medical College, HUST.

### Preparation of ^18^F-FHBG and microPET/CT imaging

A Siemens Inveon Acquisition Workplace (Siemens Preclinical Solution, Knoxville, TN) was used for microPET/CT imaging. After transplantation, rats were injected with ^18^F-FHBG (500±51 µCi) via the tail vein at days 2, 3 and 7, and prone position microPET/CT imaging was performed after 1 h for 20 min. The standard ordered-subset expectation maximization method was used for microPET image reconstruction. CT images were used for both attenuation correction of emission data and image fusion. Fusion of microPET/CT images showed horizontal, coronal and sagittal sections. Then, regions of interest (ROIs) were manually drawn. Quantitative analysis of ^18^F-FHBG uptake in the brain, heart, liver, lung, kidney, stomach, intestine and spleen was performed.

### Bioluminescence imaging

A Maestro small animal imaging system (Cambridge Research & Instrumentation) was used for bioluminescence imaging. Rat models received an abdominal injection of 200 µl firefly luciferase substrate (10 mg/ml D-Luciferin; Xenogen, Alameda, CA). Bioluminescence imaging was performed for 5 min after injection. The size of the cooled charge coupled device (CCD) was 4×4 inches, and the operating temperature was –70°C. Bioluminescence imaging was performed at days 2, 3, 5, 7, 10 and 15 after transplantation.

### Fluorescence imaging

A fluorescence imaging system for living animals (Roper Scientific, USA) was used. Rats (n = 3) were euthanatized at days 2, 3 and 7 after transplantation, respectively. The hair, muscle and ribs were removed to expose the thoracic cavity. The 488-nm excitation and 510-nm emission filters were used. The imaging time was 500 ms. Lumazone software was used for imaging and image analysis. Region of interests (ROIs) were manually drawn for quantitative analysis of the heart region. Three control rats were also used in fluorescence imaging.

### PCR and immunohistochemistry

Total RNA was extracted from ex vivo myocardial tissue and reverse transcribed into cDNA. PCR primers for TGF were: forward, 5′-ATGCCCACGCTACTGCGG-3′; reverse: 5'-TCAGTTAGCCTCCCCCATCTC-3′. The PCR product size was 837 bp, and PCR conditions were 94°C for 5 min, followed by 35 cycles of 94°C for 30 s, 56°C for 30 s and 72°C for 1 min. The primers were provided by Invitrogen (Shanghai, China).

A portion of the ex vivo myocardial tissue was fixed with 4% formaldehyde, embedded in paraffin, and cut into 3 μm sections for hematoxylin and eosin (H&E) staining and immunohistochemistry analysis. An anti-HSV1-tk antibody (Santa Cruz, CA) was added to sections, followed by incubation at 4°C for 24 h. Then, a secondary antibody was added, followed by DAB staining, hematoxylin counterstaining, dehydration, and mounting. The stained sections were observed under an optical microscope and photographs were obtained.

The remaining ex vivo myocardial tissue was frozen in dry ice and cryosectioned into 15 μm sections. Sections were stained with anti-CD45 and anti-CD90 (eBioscience, San Diego, USA) antibodies at 4°C for 24 h, followed by incubation with a Cy3-labelled secondary antibody in the dark at room temperature for 1 h. Then, sections were counterstained with Hoechst 33258 to visualize cell nuclei, mounted with neutral glycerol, observed under a fluorescence microscope and photographed.

### Data analysis

Data were expressed as mean ± standard deviations. SPSS 13.0 software was used to analyze the data. Wilcoxon rank test for two independent samples were used for comparison between two samples. *P* <0.05 was considered significant.

## Results

**Figure 1:**
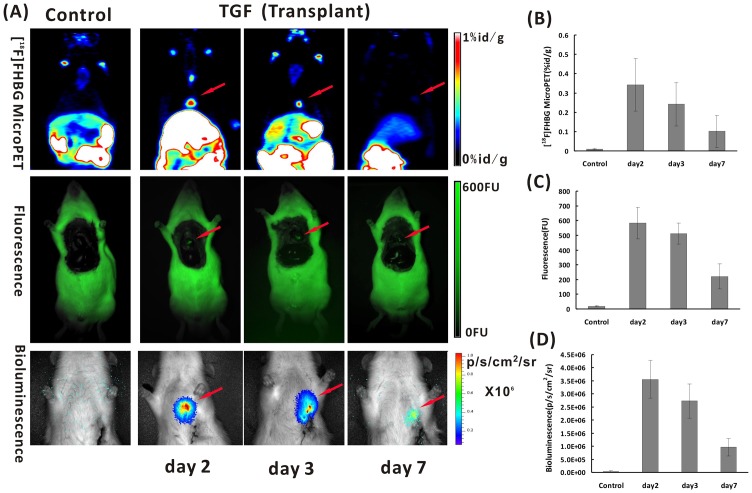
Multimodality molecular imaging in acute myocardial infarction rats after transplanted Ad5-TGF-transfected bone marrow mesenchymal stem cells (BMSCs) into myocardium. A: From images of microPET (upper row), Fluorescence (middle row) and Bioluminescence (lower row), signals in the heart region could be clearly seen in different imaging modalities (indicated by red arrows) at day 2, 3 and 7 after transplantation of Ad5-TGF-transfected BMSCs into myocardium. Semi-Quantitative analysis results obtained by regions of interest (ROIs) analysis of the region of heart from ^18^F-FHBG microPET (B), Fluorescence (C) and Bioluminescence (D) imaging shows that significant difference could be seen between the experimental group with transplanted Ad5-TGF-BMSCs and the control group with transplanted uninfected BMSCs (*P* < 0.05) in all different imaging modalities.

A serial of images of microPET, Fluorescence and Bioluminescence (Figure 1A) were obtained in acute myocardial infarction rats after transplanted Ad5-TGF-transfected BMSCs into myocardium at day 2, 3 and 7. Signals in the heart region could be clearly seen in different imaging modalities, whereas no signal could be found in the control group which transplanted with uninfected BMSCs (Figure 1A). Semi-Quantitative analysis results (Figure 1B, C and D) obtained by ROIs analysis of the heart region shows significant difference between the experimental group and the control group (*P* < 0.05) in all different imaging modalities.

### MicroPET/CT imaging

**Figure 2:**
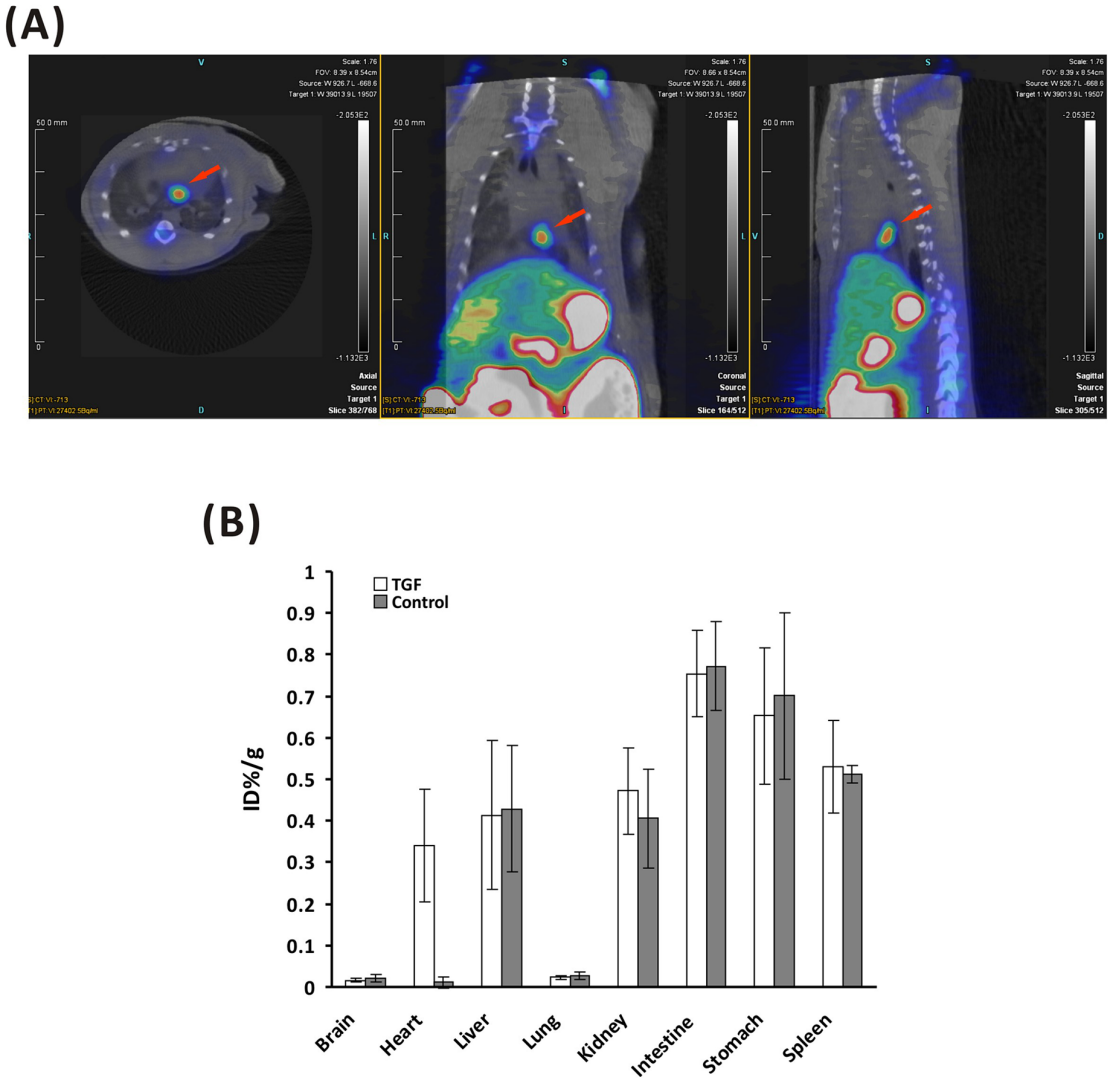
MicroPET/CT fused images in myocardial infarction rats transplanted with Ad5-TGF-transfected BMSCs at 2 days after transplantation (from left to right: horizontal, coronal and sagittal views). High signals (red arrow) could be clearly seen in the region of heart. B: Semi-quantitative analysis of ^18^F-FHBG uptake in different organs from the images. Significant difference exists in the heart between the experimental rats which were transplanted of Ad5-TGF transfected BMSC into myocardial infarction area and the control rats with non-transfected BMSCs only (n = 5, *P* < 0.05). However, no significant difference exists in other organs.

MicroPET/CT was performed on modeled rats with transplanted BMSCs at 2 days after transplantation. The fusion image in Figure 2A shows obvious ^18^F-FHBG uptake in the myocardial infarction region of rats transplanted with Ad5-TGF-infected BMSCs. Next, quantitative analysis was performed. As shown in Figure 2B, ^18^F-FHBG uptake in the brain, heart, liver, lung, kidney, intestine, stomach and spleen of the modeled group was 0.016±0.005, 0.341±0.136, 0.413±0.179, 0.023±0.004, 0.472±0.104, 0.754±0.104, 0.653±0.164 and 0.531±0.112 percentage of injected dose per gram (%ID/g) (n = 5), respectively, while it showed 0.021±0.008, 0.011±0.014, 0.429±0.151, 0.028±0.009, 0.406±0.119, 0.772±0.107, 0.701±0.201 and 0.512±0.021%ID/g, respectively in the controlled group (n = 5). The heart/lung ratio of ^18^F-FHBG uptake of the modeled group was 31-fold higher than that of the negative control group (*P*  =  0.043). Next, monitoring was performed for 1 week. As shown in Figure 1A and 1B, ^18^F-FHBG uptake in the heart region of the modeled group at days 3 and 7 after transplantation was 0.241±0.112 and 0.101±0.082%ID/g, respectively.

### Bioluminescence imaging

**Figure 3:**
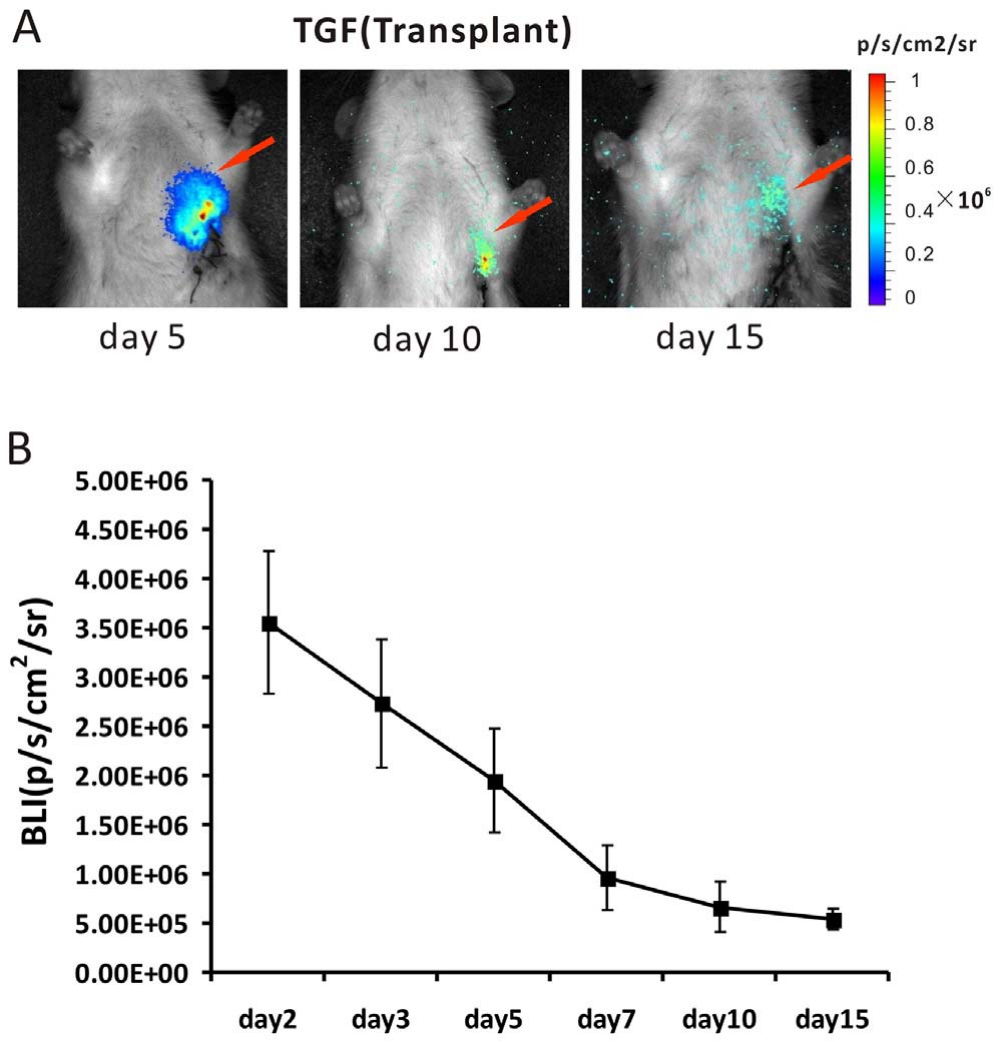
Continuous bioluminescence images after different days of transplanted Ad5-TGF transfected BMSCs into myocardial infarction rats (A). The red arrows indicate the signals in the heart region. B: Semi-quantitative analysis of images shows that the signal decreased with the time after transplantation (n = 5).

Continuous monitoring of transfected stem cells was performed for 2 weeks by bioluminescence imaging of the same group of the myocardial infarcted rats that had already been scanned by microPET/CT (Figure 3A). Quantitative analysis at days 2, 3, 5, 7, 10 and 15 showed that the intensity of the bioluminescence signal in the heart region of rats in the modeled group was (3.556±0.725)×10^6^, (2.731±0.652)×10^6^, (1.946±0.531)×10^6^, (0.962±0.326)×10^6^, (0.662±0.266)×10^6^ and (0.442±0.126)×10^6^ photons/s/cm^2^/sr, respectively (Figure 3B) (n = 5). As a comparison, the intensity of the optical signal was only (0.033±0.03)×10^6^ photons/s/cm^2^/sr in the heart region of rats in the negative control group (data not shown).

### Fluorescence imaging

Continuous monitoring was also performed for 1 week by fluorescence imaging of transplanted BMSCs in myocardial infarcted rats. Fur, muscle and ribs were removed to expose the thoracic cavity (Figure 1A). Visible green fluorescence was detected in the heart region of rats in the modeled group, whereas no visible fluorescence was detected in the negative control group. Quantitative analysis showed that the fluorescence intensity in the heart region of rats in the modeled group at days 2, 3 and 7 was 582±107, 512±71 and 221±85 FU, respectively (n = 3). However, only 19±5 FU was measured in the heart region of rats in the negative control group (n = 3).

### PCR and immunohistochemistry

**Figure 4:**
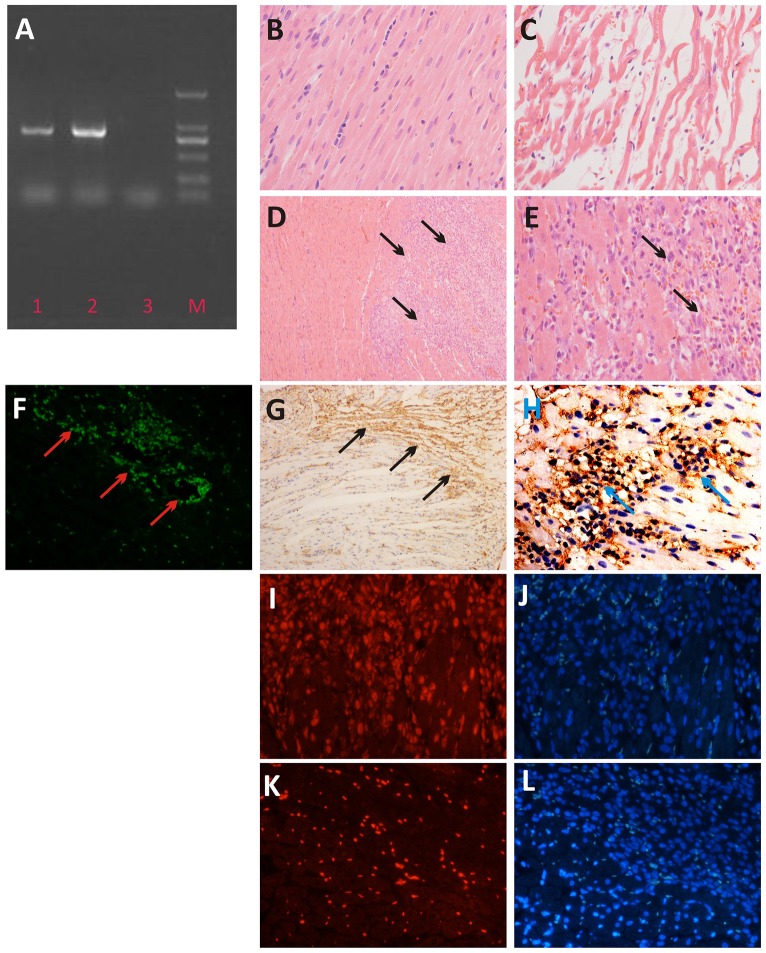
In vitro analysis of the heart tissue after transplanted with Ad5-TGF-transfected BMSCs or non-transfected BMSCs. A: PCR result: 1. rat heart tissue transplanted with Ad5-TGF-transfected BMSCs; 2. Ad5-TGF-transfected BMSCs, 3. the control rat heart tissue with transplanted with non-transfected BMSCs; M, marker (from lower to upper: 100, 250, 500, 750, 1000 and 1500 bp). B: H&E staining of the heart tissue in healthy rats. C: H&E staining of the rat heart tissue with myocardial infarction; D and E: H&E staining of the rat heart tissue with BMSCs transplantation (BMSCs are indicated by black arrows, 100× and 400×, respectively); F: Frozen section of a rat in the modeled group with rBMSC transplantation (eGFP expression is indicated by red arrows); G and H: Immunohistochemistry of HSV1-tk in the heart tissue of rats in the modeled group with BMSC transplantation (brown indicates positive staining, 100× and 400×, respectively). I and K: Immunofluorescence of Cy3-CD90 and Cy3-CD45 in the heart tissue of rats in the modeled group with BMSC transplantation (CD90 is shown as red, CD45 was negative, 400×). J and L: Immunofluorescence of I and K with nuclear counterstaining (blue, 400×).

After PCR and electrophoresis, specific DNA bands were detected between 750 and 1000 bp in DNA obtained from both the rat myocardial tissue and TGF-transfected BMSCs of the modeled group (Figure 4A). The DNA bands of cells were brighter than those of the tissue. No DNA bands were detected in the DNA from myocardial tissue of rats in the negative control group. H&E staining of myocardial tissue showed that Normal myocardial fibers displayed a regular and diffuse distribution (Figure 4B); however, infarcted myocardial fibers were swollen, disorganized, contained vacuoles and even broken (Figure 4C). Transplanted BMSCs distributed in the myocardial tissue gap were obvious, as shown in Figure 4D and E. eGFP fluorescence from transplanted BMSCs in the infarcted myocardium of rats in the modeled group was observed under a fluorescence microscope (Figure 4F). Positive staining (brown) of transplanted TGF-transfected BMSCs was detected by immunohistochemistry with an anti-HSV1-tk antibody and DAB staining (Figure 4G and H). No obvious staining was detected in normal myocardial cells. Immunofluorescence staining showed that transplanted BMSCs were positive for Cy3-CD90 (red, Figure 4I), but negative for Cy3-CD45 (Fig. 4K). Nuclei were stained and represented in blue (Figure 4J and 4L).

## Discussion

This study has shown that microPET/CT, fluorescence and bioluminescence imaging are non-invasive techniques that can be used to repeatedly monitor transplanted stem cells in animal models of myocardial infarction. We performed microPET/CT, fluorescence and bioluminescence imaging on each animal model of myocardial infarction at days 2, 3 and 7 after transplantation. Images of the transplanted region of the heart were even obtained by BLI at 15 days after transplantation. The semi-quantitative analyses of TGF expression obtained by the three imaging techniques were changing at the same trend over time. Finally, we verified the imaging results with the ex vivo assays using PCR and histological identification of the stem cell transplanted heart tissue. This study is the successful application of three different molecular imaging techniques to monitor transplanted stem cells in vivo in a myocardial infarction model.

Because stem cell transplantation is a valid treatment for ischemic heart disease, non-invasive molecular imaging methods have been actively pursued to monitor transplanted stem cells. First, PET reporter gene imaging is one of the most promising non-invasive molecular imaging tools, which is reliable and objective for locating transplanted stem cells in the myocardium of small animals and for quantitative analysis [12], [19], [20]. Willmann et al applied clinical PET to image large animals such as pigs, in which transplantation of human mesenchymal stem cells into the pig myocardium showed the feasibility of reporter gene imaging [21]. Subsequently, multimodality molecular imaging has been gradually developed and used to monitor transplanted stem cells in the myocardium. Higuchi et al monitored rat cardiac transplantation cell survival and positioning with both PET and MRI [11]. In a study by Wu et al [12], Fluc- and HSV1-sr39tk-transfected embryonic rat H9c2 cardiomyoblasts were transplanted into the myocardium of healthy mice, and in vivo monitoring was performed for 2 weeks using PET and BLI. However, these previous reports all used normal animals and are not an accurate reflection of stem cell survival in a lesioned environment. In this study, the major advantage is the success of continuous multimodality monitoring of stem cells in animal models of myocardial infarction, which is more intuitive and provides a reliable foundation for further applying biological therapy such as stem cells treatment in the future.

Using longitudinal monitoring with the three imaging techniques, we confirmed that BMSCs survived in lesions and did not migrate after transplantation. Based on quantitative analyses, we found that the signals in the heart region decreased as the monitoring time increased using the three imaging techniques. The signal intensity attenuated within 1 week, and by the second week the signal detected by microPET and fluorescence imaging was significantly reduced and could not be detected. Interestingly, BLI is highly sensitive, and in the second week the signal could only be detected by BLI. In our subsequent analyses, we evaluated the transplanted stem cells in the infarcted myocardium in which the local blood supply mechanism was significantly different from that in the normal myocardium. There may have been insufficient blood supply in the transplantation region, as well as the presence of lesions and inflammation, which could result in the death of some transplanted BMSCs in the infarcted region. The use of this infarction model is also the major difference compared with the normal rat study of Wu et al [12], which indicated that the survival of transplanted stem cells in the infarcted region was affected by the lesioned environment to a certain extent. One thing to note is that adenovirus was used as the TGF carrier, and it cannot insert the TGF fusion gene into the genome of BMSCs, resulting in the gradual reduction of exogenous proteins as a result of cell metabolism and proliferation.

We used the multi-functional reporter gene TGF for multimodality molecular imaging to monitor transplanted BMSCs for the treatment of ischemic heart disease. First, we combined microPET and CT technologies in which microPET provided functional imaging and CT provided accurate anatomical localization. As shown in Figure 2B, we precisely located the stem cell transplantation region by coronal, sagittal and cross sections. In the development of molecular imaging, regular conventional imaging has become an inseparable complement. We believe that in the future, PET/CT will be more applicable to clinical development of stem cell tracking techniques in vivo. Second, the sensitivity of BLI reaches a concentration of 10^–15 ^mol, which is significantly superior to that of PET [22]. During the 2 weeks of monitoring in our study, PET and fluorescence imaging could only obtain images of the transplanted rats in the first week after cell transplantation, whereas BLI was able to monitor cells for the whole duration. However, the bioluminescence technique is limited in terms of the spatial resolution by the influence of light scattering, and the penetration of the optical signal is only 2 cm [23], [24], which is consistent with the images obtained in our study (Figures 1A and 3A). Thus, BLI has limited clinical use, and it is more suitable for small animal studies. Finally, owing to tissue attenuation and refraction, the eGFP of fluorescence imaging is only 2 mm [25], [26]. Because of interference by the fur and tissue of rats, thoracotomy is required before fluorescence imaging, as shown in Figure 1A. Fluorescence is an autonomous property of cells, and the generation of fluorescence does not require an exogenous reaction substrate. We directly analyzed the expression of eGFP in tissue under a fluorescence microscope. Moreover, fluorescence imaging is superior to the other two imaging techniques in terms of its use for the in vitro analysis of eGFP.

In our study, although the dynamic observation of survival and migration of stem cells in the myocardium of the infarction model was successful, the duration was relatively short for in vivo monitoring of stem cell proliferation and differentiation as well as evaluation of whether cardiac function improved after stem cell transplantation for treating ischemic heart disease. A recent study has shown that a retrovirus can insert a target gene into the genome of stem cells, which may be advantageous for monitoring stem cell proliferation [27]. Most current studies of in vivo monitoring of transplanted stem cells to treat ischemic heart disease have been focused on cell survival, proliferation and migration [20], [28]–[31]. Further research of stem cell differentiation and evaluation of its treatment efficacy is needed.

BMSCs promote myocardial repair and revascularization, and currently it is one of the promising methods for treating myocardial infarction [32]–[34]. To improve the repair of infarcted myocardium by transplanted BMSCs, a combination of gene therapy and transplanted BMSCs is used in most cases. For example, after transfection with Bcl-2 [35] or PAI-1 [36], the BMSC survival rate increases. Furthermore, Ang1-tranfected BMSCs provide better remodeling of infarcted myocardium [37]. Integrin-linked kinase promotes the adhesion of BMSCs to the infarcted myocardium [38]. Reporter gene imaging is mature and used for in vivo monitoring regardless of whether a therapeutic gene is expressed or not, the extent of expression and the duration of therapeutic gene expression [39]. In addition, owing to the characteristics the reporter gene technique, namely good specificity and a true reflection of the stem cells, such a technique is relatively mature for in vivo monitoring of stem cell therapy [40]. Therefore, TGF reporter gene imaging is likely to be a comprehensive method not only for tracking stem cells, but also for monitoring the gene expression in combination with gene therapy, which provides a multi-faceted platform for in vivo monitoring of transplanted stem cells for treating ischemic heart diseases.

## Conclusion

This is the first application of TGF-transfected BMSC transplantation into the myocardial infarction model. Moreover, it proves that the dynamic situation of BMSCs in vivo can be monitored by microPET/CT, fluorescence and bioluminescence multimodality imaging. This study indicates that TGF can be used for in vivo monitoring of transplanted BMSCs for the treatment of ischemic heart disease as a multimodality reporter gene.
